# Characterization of genome-wide SNPs for the water flea *Daphnia pulicaria* generated by genotyping-by-sequencing (GBS)

**DOI:** 10.1038/srep28569

**Published:** 2016-06-27

**Authors:** Joaquín Muñoz, Anurag Chaturvedi, Luc De Meester, Lawrence J. Weider

**Affiliations:** 1 Doñana Biological Station (CSIC), Isla de La Cartuja, Av. Américo Vespucio S/N, 41092-Seville, Spain; 2 Department of Biology, Program in Ecology and Evolutionary Biology, The University of Oklahoma, 730 Van Vleet Oval, Norman, OK 73019, USA; 3 Laboratory of Aquatic Ecology, Evolution and Conservation, University of Leuven, Ch. Deberiotstraat 32, Leuven 3000, Belgium

## Abstract

The keystone aquatic herbivore *Daphnia* has been studied for more than 150 years in the context of evolution, ecology and ecotoxicology. Although it is rapidly becoming an emergent model for environmental and population genomics, there have been limited genome-wide level studies in natural populations. We report a unique resource of novel Single Nucleotide Polymorphic (SNP) markers for *Daphnia pulicaria* using the reduction in genomic complexity with the restriction enzymes approach, genotyping-by-sequencing. Using the genome of *D. pulex* as a reference, SNPs were scored for 53 clones from five natural populations that varied in lake trophic status. Our analyses resulted in 32,313 highly confident and bi-allelic SNP markers. 1,364 outlier SNPs were mapped on the annotated *D. pulex* genome, which identified 2,335 genes, including 565 within functional genes. Out of 885 EuKaryotic Orthologous Groups that we found from outlier SNPs, 294 were involved in three metabolic and four regulatory pathways. Bayesian-clustering analyses showed two distinct population clusters representing the possible combined effects of geography and lake trophic status. Our results provide an invaluable tool for future population genomics surveys in *Daphnia* targeting informative regions related to physiological processes that can be linked to the ecology of this emerging eco-responsive taxon.

High-throughput sequencing methodologies have opened up exciting possibilities in the fields of population genetics, conservation and evolutionary biology by enabling the assessment of genetic variation at genome-wide scales. The genotyping-by-sequencing strategy (GBS)^1^ is a next generation sequencing (NGS)-based method providing a cost effective approach to reduce genome complexity using restriction enzymes, being currently applied in numerous species^2^. In such a way, one can sequence part of the whole genome and generate hundreds or thousands of Single Nucleotide Polymorphisms (SNPs), providing a direct way to characterize bi-allelic molecular markers with a more-or-less uniform distribution throughout the genome. These improvements in methods and bioinformatics tools are promoting the shift from the use of few/tens of microsatellite nuclear markers to analyses of massive SNP markers in specific variable genomic regions^3^.

The purpose of this study was to apply the GBS approach to generate and characterize a genome-wide SNPs map in the water flea *Daphnia pulicaria* (Forbes 1893), a keystone aquatic herbivore inhabiting many lakes across the Nearctic. *Daphnia pulicaria* is a member of the *D. pulex* species complex, being considered as the “lake *pulex*” because of its closely-related sister taxon *D. pulex*, which inhabits small ponds. *Daphnia pulex* was the first crustacean to have its whole genome sequenced^4^. *Daphnia* species complexes have been the subject of numerous genetic studies^5^,^6^,^7^,^8^,^9^, and, despite the use of relatively low numbers of molecular markers (microsatellites and/or SNPs), key ecological and evolutionary insights (e.g., behaviour in presence of predator, response to anthropogenic impacts) on local and environmental adaptation have been revealed^10^,^11^,^12^.

*Daphnia* are phosphorus (P) rich crustacean herbivores, considered to be P-limited in natural habitats, and play a major role in whole-lake energy and phosphorus cycling^13^ (and references therein for an ecological stoichiometry review). In this way, P-supply to *Daphnia* in their habitat (ponds or lakes) seems to have an effect on this organism at several levels such as life-history variation and phenotypic plasticity^14^, physiological adjustments^15^, ribosomal RNA and DNA structure^16^, and genomic responses (e.g., gene expression)^13^,^17^.

Here, we generated a set of SNPs covering the whole *D. pulicaria* genome from clonal lineages isolated from five natural (lake) populations that varied in trophic (i.e., nutrient) status. By using these populations with different phosphorus load/supply (i.e., different eutrophication status ranging from 21 to 220 μg/L of P), we characterized outlier SNPs (i.e., particular variable positions with differentiation among populations higher than average that are likely candidates to be affected by natural selection) and mapped them to the *D. pulex* annotated genome. Our results revealed 294 outlier SNPs that play a role in important metabolic pathways potentially involved in phosphorus use and processing, ranging from cellular to ecosystem levels.

The development and characterization of hundreds of SNPs with a regular distribution across the genome will provide an invaluable genetic resource in this emergent model organism, and should give us the option to screen regions under selection. This new methodological approach will allow us to face challenges such as identifying the mechanisms and processes related to the genetic basis of adaptation in natural populations^18^.

## Results

### SNP calling and mapping

**Figure 1:**
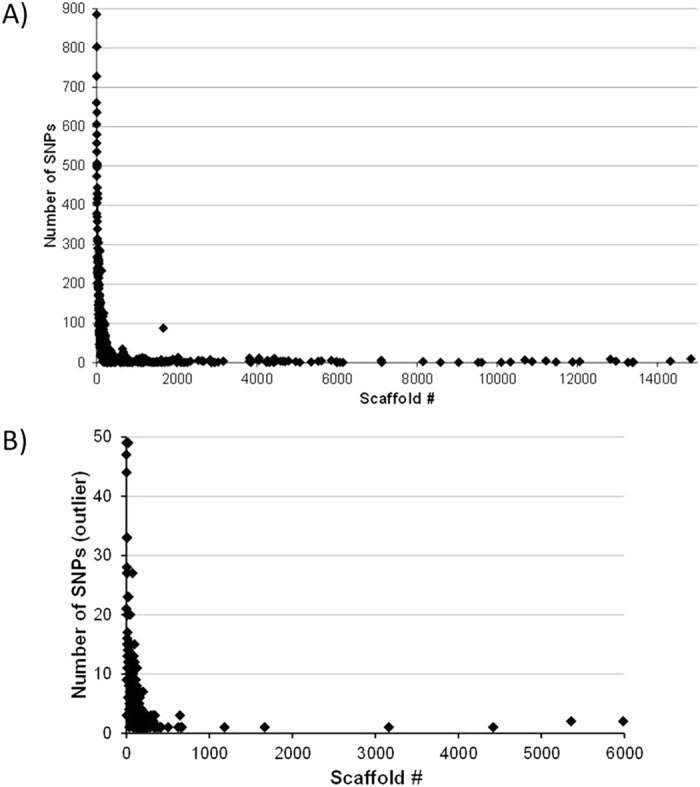
Distribution of called SNPs along the 9,080 scaffolds described on the current *Daphnia pulex* genome reference. (**A**) Representation of the total number of SNPs (i.e., 32,313). (**B)** Representation of mapped and annotated outlier SNPs (i.e., 1,364).

A total of 159 900 538 good barcoded reads (ranging from 571 683 up to 31 600 240) were obtained for the 53 clones/samples analysed, resulting in 57 440 139 tags (i.e., contigs containing the same sequence). Despite mapping short reads against a different reference genome that can limit identification of DNA variants, we detected 32,313 SNPs by mapping our reads onto the *Daphnia pulex* genome after filtering by biallelic and highly confident positions, and without linkage disequilibrium (LD) within a scaffold. Finally, we removed those SNPs with expected heterozygosity (*He*) greater than 0.5 since they might represent paralogous regions (i.e., gene duplications). Confident SNPs were included in a total of 476 scaffolds out of 9,080 described for the *D. pulex* reference genome (see Fig. 1A). The *D. pulex* reference genome is available in scaffolds with different sequence lengths (ranging from 1,000 bp to 4.1 Mb), and the number of SNPs per scaffold depends on scaffold length. We found 93.84% of SNPs (i.e., 30,323) in the first 220 scaffolds, whose individual length is greater than 100 Kb.

### Population analyses of genotyping results

**Figure 2:**
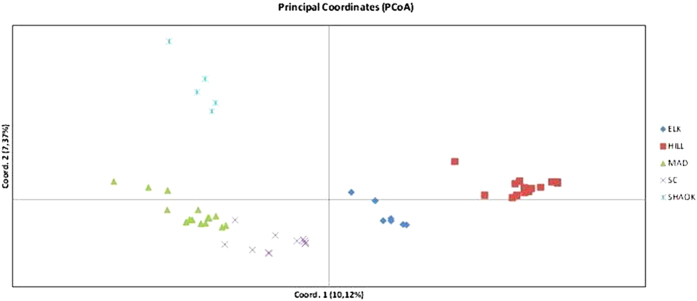
Principal Coordinate Analysis (PCoA) run on GenAlEx for 8,190 random SNPs out of the 32,313 total SNPs found in our study. Population codes are listed in Table 1.

**Figure 3:**
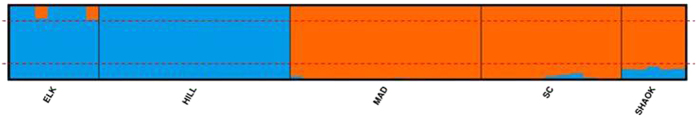
Plot of the Bayesian clustering analysis supported in STRUCTURE after computing posterior likelihood values for K = 1 to K = 6 for 53 clones on 32,313 SNPs. Two genetic clusters (i.e., K = 2) were estimated by the ΔK method of Evanno *et al*.^19^, regardless of the admixture model applied. Red dotted lines indicate the arbitrary 20% threshold for assignment to belong to a particular genetic cluster. For population/lake codes, please, refer to Table 1.

Analysis of highly confident SNPs for 53 samples performed with PCoA showed five clusters corresponding to the five lakes sampled (Fig. 2). On the other hand, the STRUCTURE clustering analysis estimated that two genetic clusters (i.e., K = 2) were most likely, as determined by the ΔK method of Evanno *et al*.^19^. Our plot (Fig. 3) showed that the two genetic clusters correspond to northern and southern populations (i.e., HILL and ELK – North; SC, MAD, and SHAOK – South).

### Description of outlier SNPs (gene annotation and role in metabolic pathways)

1,499 outlier SNPs were detected by the Lositan programme (only 1,364 SNPs could be mapped on to the annotated *D. pulex* reference genome) along 212 different scaffolds (Fig. 1B). The Bayescan analysis found a lower number of outlier SNPs, which were represented in the list obtained from Lositan. Potentially adaptive SNPs identified by Lositan resulted in 2,335 *Daphnia pulex* official JGI_V11 ID annotated genes (according to wFleaBase - dpulex1_JGI_V11_annotatedgene.gff.gz), from which 401 genes contained at least one outlier SNP position. The number of SNPs within gene ID ranged from one to six (see Appendix 1 for details).

**Figure 4:**
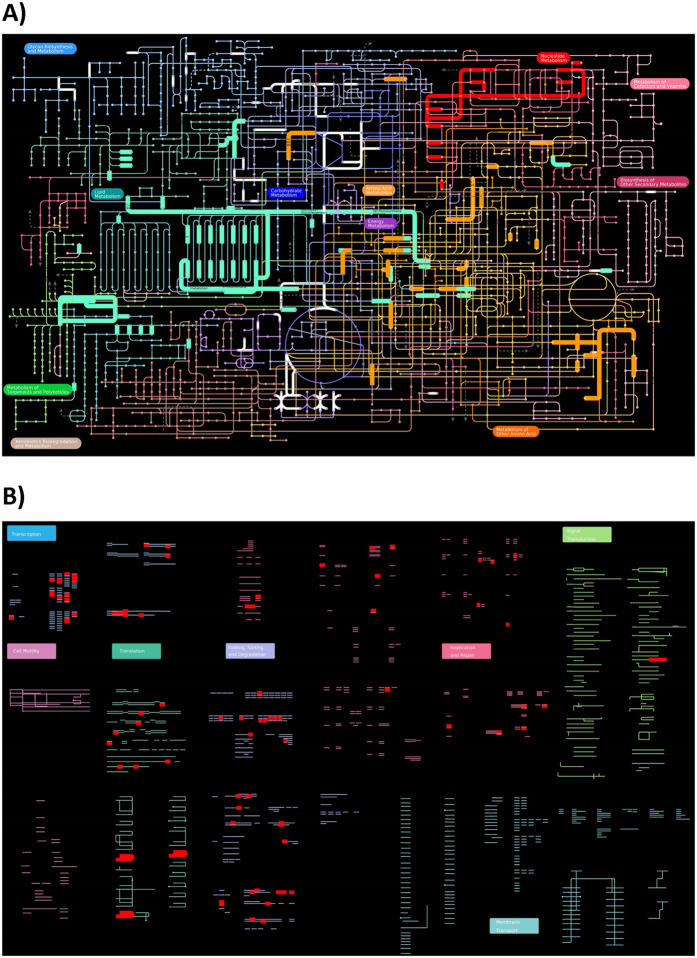
Plots of the annotated outlier SNPs found on different pathway maps as implemented by iPath2 46,47. (**A**) Metabolic pathways highlighting the three main ones where our outlier SNPs are involved: Lipid metabolism (green colour), Amino Acid metabolism (orange colour), and Nucleotide metabolism (red colour). Pathways indicated in white colour (and narrower lines) show the rest of outlier SNPs; (**B**) Regulatory pathways highlighted in red. Note that no gene/locus is represented on three key regulatory paths: cell mobility, membrane transport, and signal transduction.

Plots obtained from iPATH v2 resulted in 294 KOG genes associated mainly with three metabolic pathways (172 genes have a role in the metabolism of nucleotides, amino acids and lipids; Fig. 4A) and four regulatory pathways (122 genes have a role in transcription, translation, replication and repair, and folding, sorting and degradation; Fig. 4B).

## Discussion

Genomic resources have increased our ability to study the evolutionary ecology and population genetic structure of the *Daphnia pulex* species complex in more detail in recent years^4^, but can be greatly improved upon by NGS technologies currently available or soon-to-be available in the near future^20^. Using GBS methodology on genomic DNA from 53 samples/clones of *D. pulicaria*, we developed the first whole-genome resources for this important widely-distributed Nearctic lake species. Our dataset presents complementary information that can be compared with recent studies reporting differential gene expression in the transcriptome of *D. pulex* in response to phosphorus supply^13^,^17^. A production of high-quality SNPs was achieved, analysed and illustrated by different approaches, obtaining information on annotations blasted onto the current *D. pulex* reference genome. These GBS-derived genomic resources provide valuable molecular resources for high-resolution linkage mapping, association analysis, and gene verification, which will be of great use to better understand demographic, adaptive and micro-evolutionary processes in the keystone and emergent eco-responsive model organism, *Daphnia*.

Population genetic differentiation among populations of passively dispersed aquatic invertebrates in most cases has been found to be strong, despite the potential dispersal capacity facilitated by water birds and other vectors transporting their diapausing eggs^21^,^22^,^23^. In *Daphnia*, where diapausing eggs are enclosed in the protective ephippium, population genetic differentiation has been observed even at small/medium (i.e., less than 1 Km) spatial scales^24^,^25^. Our results are in agreement with a highly structured population model (Fig. 2) across the whole genome (i.e., 8,190 SNPs). This would indicate a low likelihood of colonization by novel genotypes/clones in already established populations, which has been reported in previous studies^26^,^27^,^28^. However, our inference of population structure using a Bayesian approach (i.e., STRUCTURE program) indicates a co-ancestry relationship with clustering of clones into two main lake groups: (i) Hill Lake and Elk Lake, and (ii) Madison Lake, Shaokotan Lake, South Center Lake (Fig. 3). These results could be indicative of a geographic and/or an environmental association. One cluster includes the two northern lakes (Hill and Elk), whilst the other cluster includes the three southern ones. In the present study, we cannot reject an isolation-by-distance effect due to a historical colonization event, but a relationship between genomic response and ecological traits cannot be ruled out since both Hill and Elk lakes are located in a different ecoregion, when compared to the other three lakes (South Center, Madison, and Shaokotan). In addition, the phosphorus (P)-loadings of Hill and Elk lakes are on the lower end of the trophic state range for the five lakes sampled (see Table 1), which could also be affecting the genetic clustering relationships noted. Preliminary analyses performed in *D. pulicaria* sampled from one of our five lakes (South Center), using hyper-variable nuclear markers (i.e., microsatellites), support clear evolutionary consequences of anthropogenic environmental change (i.e., change in P-supply) on population structure through time^12^. These results are consistent with previous reports indicating that *Daphnia* populations show genotype x environment interactions when subjected to different P-conditions. As a consequence, different clones/genotypes are sensitive to P-availability^15^,^29^, which can include the differential expression of hundreds of genes^13^,^17^ when subjected to nutritional environments that vary in P-supply.

Our results indicate that the outlier (and potentially adaptive) loci found might, amongst others, have important roles in phosphorus processing including assimilation and maintenance (see Fig. 4A,B). In fact, evidence suggests that variation in the environmental supply of P affects expression of highly conserved genes^15^ (for a review). It has also been reported that phosphorus supply drives rapid turnover of membrane phospholipids in phytoplankton^30^. Particularly, in *Daphnia* it has been shown that under low quality food (i.e., high carbon – C to low phosphorus – P ratio: C:P), this organism can maintain P-homeostasis by reducing C-uptake and/or increasing P-retention, which is in agreement with stoichiometric theory^31^. Additionally, it has been suggested (and assessed) that the role of organismal C:P ratios may be functionally related to the structural and copy number variation of ribosomal-RNA genes^16^. Recent studies have reported large differences in gene expression levels under different P-diets in *Daphnia* at the intra-specific level^17^,^31^. These previous studies have found hundreds of differentially expressed genes in a comparison between genotypes run under low and high P environmental conditions. Important KEGG (i.e., Kyoto Encyclopedia of Genes and Genomes) pathways involving differentially-regulated genes have been noted for glycogen synthesis and lipid metabolism, among others. Our results, at the genomics level, indicate that some functional genes annotated on the current *Daphnia pulex* reference genome, might have an important role in lipid metabolism, mainly when considering exclusive genes involved directly in metabolic pathways (i.e., removing those genes linked to both metabolic and regulatory pathway; see Suppl. Figure). Whereas none of the outlier SNPs involving functional genes are related to three main regulatory pathways (cell mobility, membrane transport or signal transduction; see Fig. 4B), they are highly represented on regulatory pathways such as transcription, translation, replication and repair, and folding, sorting and degradation.

The new genomic resources reported in our study include parts of the genome that appear to be less affected by selection, and thus, may prove useful for future demographic studies. In addition, we found signatures of selection for candidate genes (see Appendix 1) related to abiotic stressors that will help to unravel adaptive molecular processes related to variation in environmental conditions such as lake eutrophication. Future analyses on these and other gene annotations should shed light on the selective forces structuring, at the genome-wide level, different genotypes and natural populations influenced by environmental pressure. Additionally, our resources and results may improve our understanding of how the genome interacts with the environment to produce the diversity of phenotypes found at the intra-specific level.

Future work that integrates molecular markers, ecological traits and geographic parameters will be necessary to shed light on the effects of both abiotic and biotic environmental factors as selective forces impacting the population genetic structure in *Daphnia* species complexes. In our case, we have identified candidate genes that can be used in the near future to study/manipulate natural populations in assessing physiological responses to varying nutrient environments (i.e., C:P ratios).

## Methods

### Study system and sampling

The life cycle of most daphniids is characterized by an asexual (parthenogenetic) mode of reproduction, shifting to a sexual phase at the end of the growing season when resources are scarce or environmental conditions are not favourable. During the first stages of sexual reproduction, some direct-developing (i.e., subitaneous) eggs produced by female *Daphnia* can develop into males, which are genetic replicates of the mothers, since *Daphnia* have environmental sex determination. These males can then mate with sexually-receptive females, which leads to the production of sexual (i.e., recombinant) eggs that are produced inside a scleroterized structure termed an ephippium. When the mother molts, the ephippium is shed into the water column, where it either sinks to the bottom sediments or rafts to the shoreline. Ephippia serve as both a diapausing stage as well as a (passive) dispersal stage, which increases the likelihood for population survival during harsh environmental conditions. Ephippial (diapausing) eggs will hatch when favourable conditions appear in natural populations. However, isolated resting eggs (i.e., different clones from sexual reproduction) can be hatched under laboratory conditions (i.e., resurrection ecology) allowing one to address studies on population genetic structure, evolutionary biology and adaptive response to environmental changes^12^,^32^,^33^,^34^.

**Figure 5:**
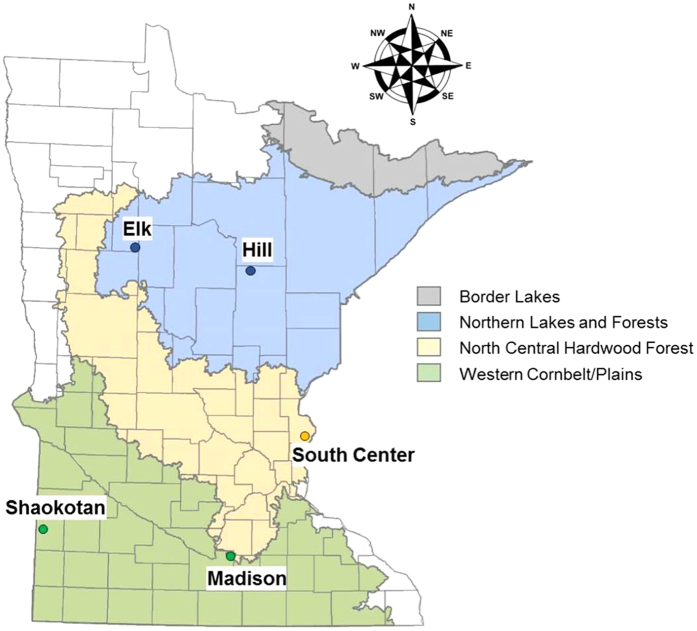
Location of lakes sampled and analysed from Minnesota, USA. Four different ecoregions (grey, blue, yellow and green colour areas) are classified by MN Pollution Control Agency, and in this study we have lakes found in three of these ecoregions. Map was kindly provided by the Minnesota Department of Natural Resources, Section of Fisheries.

In this study, we hatched diapausing eggs from the upper sediment layers (i.e., down to a depth of ~4 cm) of five lakes located in Minnesota (USA). In addition, we established clones from live-caught animals from the water column. The lakes covered a range of trophic states from mesotrophic to hyper-eutrophic conditions. Considering phosphorus concentration ([P]) as one of the major factors involved in lake eutrophication, we covered a range from ~21 to ~220 μg/L [P] (Table 1). Additionally, the geographic distribution of the lakes covered up to three different ecoregions in Minnesota as described by the Minnesota Pollution Control Agency (Fig. 5).

### DNA extraction, genome reduction and sequencing

A total of 53 *D. pulicaria* clones from the five lakes (ranging from 5 to 15 clones per lake) were raised in separate 900 ml glass jars in the lab. DNA from ~100 pooled individuals per clone was isolated using a commercial kit (DNeasy Blood & Tissue Kit, Qiagen Inc., Valencia, CA, US). DNA concentrations were measured with a Qubit 2.0 Fluorometer (Life Technologies), with values ranging from 8 to 73 ng/μL (194–3285 ng of total DNA). Genotyping-by-Sequencing (GBS) libraries were constructed at the Institute for Genomic Diversity (IGD, Ithaca, NY, US) and sequenced on an Illumina HiSeq 2000/2500 (100 bp, single-end reads), according to Elshire *et al*.^1^, using the restriction enzyme *ApeKI* (GCWGC) for digestion. A library with unique barcodes for each clone, plus a blank sample, was created.

### SNP calling and population structure analyses

The GBS analysis pipeline (Tassel ver. 4.0), an extension of the Java program TASSEL^2^,^35^, was used to call SNPs from the sequenced GBS library (.fastq rawdata set; see Appendix 2). Tags were aligned to the *Daphnia pulex* reference genome wfleabase.org/genome/Daphnia_pulex/current/fasta/dpulex-all-chromosome-jgi060905.fasta.gz, which contains 9,080 scaffolds. A variant calling format (VCF) file was filtered for variant sites to cover at least 90% of individuals and for biallelic positions.

Linkage disequilibrium (LD) between pairs of variant sites was evaluated by using the software package TASSEL (LD type: Full Matrix^35^), and estimated by using standardized disequilibrium coefficients (*D’*^36^) and squared allele-frequency correlations (*r*^*2*^ 37). The probabilities of obtaining LD estimates at least as extreme as those observed under a hypothesis of linkage equilibrium (*p*-values) were calculated by using Fisher’s exact test for site pairs with two alleles each. *P*-values ≤ 0.05 were considered significant to remove all but one linked SNPs for further analyses.

Population genomic analyses were performed as follows. First, we ran a principal coordinate analysis (PCoA) using the codominant genetic distance matrix generated from highly confident SNPs to elucidate the genetic relationships for the geographic area sampled using GenAlEx ver. 6.501^38^,^39^. Second, the extent of admixture and the origin of the different genetic proportions were investigated using a Bayesian clustering method implemented in the STRUCTURE ver. 2.3.4 program^40^. We calculated the posterior likelihood values for a range of clusters from K = 2 to 5. For each K, we ran four simulations with a burn-in of 30,000 Markov-Chain-Monte-Carlo (MCMC) iterations and 300,000 iterations after burn-in. We applied the correction of Evanno *et al*.^19^ to estimate the most likely value for K by using the CLUMPAK server (http://clumpak.tau.ac.il/bestK.html).

### Outlier identification and mapping annotation

We performed a genome scan analysis on all individuals to identify the potential adaptive loci, which can be directly under selection or closely-linked (i.e., “hitchhiking”) to loci under selection. We used two methods, Lositan programme^41^ and Bayescan^42^,^43^ for this purpose. Lositan uses *F*_*DIST*_^44^ based on an infinite island model of populations^45^. Bayescan uses multinomial-Dirichlet distribution and estimates posterior probabilities of a locus to be under selection or not. In Lositan, we used 100 000 simulations with neutral mean *F*_*ST*_ (force mean *F*_*ST*_) and 0.99 confidence interval. In Bayescan, we used 100 000 iterations with a sample size of 10 000 and thinning interval of 10.

Finally, we mapped the outlier SNPs to the *Daphnia pulex* genome based on the published gene models at http://genome.jgi-psf.org/Dappul/Dappul.download.ftp.html (FrozenGeneCatalog20110204.gff.gz^4^) using BEDTOOLS window feature (-w 5000), which takes into account the given number of base pairs upstream and downstream of the outlier SNP/position due to the fact that an identified SNP may not be directly under selection, but linked (“hitchhiking”) to a locus under selection, covering 10,000 base pairs in total.

Annotated genes included into the KOG_JGI nomenclature (i.e., EuKaryotic Orthologous Groups, a tool for identifying orthologous and paralogous proteins) were plotted by iPATH v2 tool^46^,^47^ (interactive Pathway Explorer 2; http://pathways.embl.de/iPath2.cgi#) resulting in the different cellular pathways (metabolic, regulatory, and biosynthesis of secondary metabolites).

## Additional Information

**How to cite this article**: Muñoz, J. *et al*. Characterization of genome-wide SNPs for the water flea *Daphnia pulicaria* generated by genotyping-by-sequencing (GBS). *Sci. Rep.* **6**, 28569; doi: 10.1038/srep28569 (2016).

## Supplementary Material

Supplementary Dataset 1

Supplementary Information

## Figures and Tables

**Table 1:** Name and geographic reference, trophic status, and phosphorus concentration of the five populations analysed in this study.

Lake name/County/N	Coordinates	Status 2008	([P]μg/L)
Elk Lake (ELK)/Clearwater/7	47°11′15″N 95°12′54″W	Mesotrophic	21
Hill Lake (HILL)/Aitkin/15	47°00′45″N 93°35′51″W	Mesotrophic	22–36
Madison Lake (MAD)/Blue Earth/15	44°11′32″N 93°48′37″W	Hyper-eutrophic	65–95
South Center Lake (SC)/Chisago/11	45°22′31″N 92°49′32″W	Eutrophic	25–50
Shaokotan Lake (SHAOK)/Lincoln/5	44°24′13″N 96°21′43″W	Eutrophic	80–220

N = Number of clones analysed in this study.

## References

[b1] Elshire R. J. . A robust, simple genotyping-by-sequencing (GBS) approach for high diversity species. PLos ONE 6(5), e19379 (2011). 21573248 10.1371/journal.pone.0019379 PMC3087801

[b2] Glaubitz J. C. . TASSEL-GBS: A high capacity genotyping by sequencing analysis pipeline. PLos ONE 9(2), e90346 (2014). 24587335 10.1371/journal.pone.0090346 PMC3938676

[b3] Helyar S. J. . Application of SNPs for population genetics of nonmodel organisms: new opportunities and challenges. Molecular Ecology Resources 11(Suppl. 1), 123–136 (2011). 21429169 10.1111/j.1755-0998.2010.02943.x

[b4] Colbourne J. K. . The ecoresponsive genome of *Daphnia pulex*. Science 331, 555–561 (2011). 21292972 10.1126/science.1197761 PMC3529199

[b5] Colbourne J. K., Robinson B., Bogart K. & Lynch M. Five hundred and twenty-eight microsatellite markers for ecological genomic investigations using *Daphnia*. Molecular Ecology Notes 4, 485–490 (2004).

[b6] Cristescu M. E. A., Colbourne J. K., Radivojac J. & Lynch M. A microsatellite-based genetic linkage map of the waterflea, *Daphnia pulex*: On the prospect of crustacean genomics. Genomics 88, 415–430 (2006). 16624519 10.1016/j.ygeno.2006.03.007

[b7] Marková S., Dufresne F., Rees D. J., Berný M. & Kotlík P. Cryptic intercontinental colonization in water fleas *Daphnia pulicaria* inferred from phylogenetic analysis of mitochondrial DNA variation. Molecular Phylogenetics and Evolution 44, 42–52 (2007). 17292634 10.1016/j.ympev.2006.12.025

[b8] Orsini L., Jansen M., Souche E. L., Geldof S. & De Meester L. Single nucleotide polymorphism discovery from expressed sequence tags in the waterflea *Daphnia magna*. BMC Genomics 12, 309 (2011). 21668940 10.1186/1471-2164-12-309 PMC3146954

[b9] Tucker A. E., Ackerman M. S., Eads B. D., Xu S. & Lynch M. Population-genomic insights into the evolutionary origin and fate of obligately asexual *Daphnia pulex*. Proceedings of the National Academy of Sciences USA 110(39), 15740–15745 (2013). 10.1073/pnas.1313388110 PMC3785735 23959868

[b10] Cousyn C. . Rapid, local adaptation of zooplankton behavior to changes in predation pressure in the absence of neutral genetic changes. Proceedings of the National Academy of Sciences USA 98(11), 6256–6260 (2001). 10.1073/pnas.111606798 PMC33455 11353872

[b11] Orsini L., Spanier K. I. & De Meester L. Genomic signature of natural and anthropogenic stress in wild populations of the waterflea *Daphnia magna*: validation in space, time and experimental evolution. Molecular Ecology 21, 2160–2175 (2012). 22257313 10.1111/j.1365-294X.2011.05429.x

[b12] Frisch D. . A millennial-scale chronicle of evolutionary responses to cultural eutrophication in *Daphnia*. Ecology Letters 17, 360–368 (2014). 24400978 10.1111/ele.12237

[b13] Jeyasingh P. D. . How do consumers deal with stoichiometric constraints? Lessons from functional genomics using *Daphnia pulex*. Molecular Ecology 20, 2341–2352 (2011). 21521393 10.1111/j.1365-294X.2011.05102.x

[b14] Jeyasingh P. D. & Weider L. J. Phosphorus availability mediates plasticity in life-history traits and predator-prey interactions in *Daphnia*. Ecology Letters 8, 1021–1028 (2005).

[b15] Jeyasingh P. D. & Weider L. J. Fundamental links between genes and elements: evolutionary implications of ecological stoichiometry. Molecular Ecology 16, 4649–4661 (2007). 17944849 10.1111/j.1365-294X.2007.03558.x

[b16] Weider L. J., Glenn K. L., Kyle M. & Elser J. J. Associations among ribosomal (r)DNA intergenic spacer length, growth rate, and C:N:P stoichiometry in the genus *Daphnia*. Limnology and Oceanography 49, 1417–1423 (2004).

[b17] Chowdhury P. R. . Differential transcriptomic responses of ancient and modern *Daphnia* genotypes to phosphorus supply. Molecular Ecology 24, 123–135 (2015). 25410011 10.1111/mec.13009

[b18] Orsini L., Andrew R. & Eizaguirre C. Evolutionary ecological genomics. Molecular Ecology 22, 527–531 (2013). 23320463 10.1111/mec.12177

[b19] Evanno G., Regnaut S. & Goudet J. Detecting the number of clusters of individuals using the software STRUCTURE: a simulation study. Molecular Ecology 14, 2611–2620 (2005). 15969739 10.1111/j.1365-294X.2005.02553.x

[b20] Mayer C., Leese F. & Tollrian R. Genome-wide analysis of tandem repeats in *Daphnia pulex* - a comparative approach. BMC Genomics 11, 277 (2010). 20433735 10.1186/1471-2164-11-277 PMC3152781

[b21] Mills S., Lunt D. H. & Gómez A. Global isolation by distance despite strong regional phylogeography in a small metazoan. BMC Evolutionary Biology 7, 225 (2007). 17999774 10.1186/1471-2148-7-225 PMC2254418

[b22] Muñoz J. . Phylogeography and local endemism of the native Mediterranean brine shrimp *Artemia salina* (Branchiopoda: Anostraca). Molecular Ecology 17, 3160–3177 (2008). 18510585 10.1111/j.1365-294X.2008.03818.x

[b23] Ventura M. . Local and regional founder effects in lake zooplankton persist after thousands of years despite high dispersal potential. Molecular Ecology 23(5), 1014–1027 (2014). 24393221 10.1111/mec.12656

[b24] Hamrová E., Mergeay J. & Petrusek A. Strong differences in the clonal variation of two *Daphnia* species from mountain lakes affected by overwintering strategy. BMC Evolutionary Biology 11, 231 (2011). 21824417 10.1186/1471-2148-11-231 PMC3161014

[b25] Yin M., Wolinska J. & Giessler S. Clonal diversity, clonal persistence and rapid taxon replacement in natural populations of species and hybrids of the *Daphnia longispina* complex. Molecular Ecology 19(19), 4168–4178 (2010). 20819161 10.1111/j.1365-294X.2010.04807.x

[b26] Louette G., Vanoverbeke J., Ortells R. & De Meester L. The founding mothers: the genetic structure of newly established *Daphnia* populations. Oikos 116(5), 728–741 (2007).

[b27] Ortells R., Vanoverbeke J., Louette G. & De Meester L. Colonization of *Daphnia magna* in a newly created pond: founder effects and secondary immigrants. Hydrobiologia 723(1), 167–179 (2014)

[b28] De Meester L., Gómez A., Okamura B. & Schwenk K. The Monopolization Hypothesis and the dispersal-gene flow paradox in aquatic organisms. Acta Oecologica 23, 121–135 (2002).

[b29] Jeyasingh P. D., Weider L. J. & Sterner R. W. Genetically-based trade-offs in response to stoichiometric food quality influence competition in a keystone aquatic herbivore. Ecology Letters 12(11), 1229–1237 (2009). 19719840 10.1111/j.1461-0248.2009.01368.x

[b30] Martin P., Van Mooy B. A., Heithoff A. & Dyhrman S. T. Phosphorus supply drives rapid turnover of membrane phospholipids in the diatom *Thalassiosira pseudonana*. ISME J. 5(6), 1057–1060 (2011). 21160536 10.1038/ismej.2010.192 PMC3131859

[b31] Chowdhury P. R., Lopez J. A., Weider L. J., Colbourne J. K. & Jeyasingh P. D. Functional genomics of intraspecific variation in carbon and phosphorus kinetics in *Daphnia*. Journal of Experimental Zoology 321A, 387–398 (2014). 24838198 10.1002/jez.1869

[b32] Weider L. J., Lampert W., Wessels M., Colbourne J. K. & Limburg P. Long-term genetic shifts in a microcrustacean egg bank associated with anthropogenic changes in the Lake Constance ecosystem. Proceedings of the Royal Society B 264, 1613–1618 (1997).

[b33] Hairston N. G. . Lake ecosystems - Rapid evolution revealed by dormant eggs. Nature 401, 446 (1999).

[b34] Caceres C. E. Interspecific variation in the abundance, production, and emergence of *Daphnia* diapausing eggs. Ecology 79, 1699–1710 (1998).

[b35] Bradbury P. J. . TASSEL: software for association mapping of complex traits in diverse samples. Bioinformatics 23, 2633–2635 (2007). 17586829 10.1093/bioinformatics/btm308

[b36] Hedrick P. W. Gametic disequilibrium measures: Proceed with caution. Genetics 117, 331–341 (1987). 3666445 10.1093/genetics/117.2.331 PMC1203208

[b37] Weir B. S. Genetic Data Analysis II (Sinauer, Sunderland, MA, 1996).

[b38] Peakall R. & Smouse P. E. GENALEX 6: genetic analysis in Excel. Population genetic software for teaching and research. Molecular Ecology Notes 6, 288–295 (2006). 10.1093/bioinformatics/bts460 PMC3463245 22820204

[b39] Peakall R. & Smouse P. E. GenAlEx 6.5: genetic analysis in Excel. Population genetic software for teaching and research-an update. Bioinformatics 28, 2537–2539 (2012). 22820204 10.1093/bioinformatics/bts460 PMC3463245

[b40] Pritchard J. K., Stephens M. & Donnelly P. Inference of population structure using multilocus genotype data. Genetics 155, 945–959 (2000). 10835412 10.1093/genetics/155.2.945 PMC1461096

[b41] Antao T., Lopes A., Lopes R. J., Beja-Pereira A. & Luikart G. LOSITAN: A workbench to detect molecular adaptation based on a Fst-outlier method. BMC Bioinformatics 9, 323 (2008). 18662398 10.1186/1471-2105-9-323 PMC2515854

[b42] Foll M. & Gaggiotti O. A genome-scan method to identify selected loci appropriate for both dominant and codominant markers: A Bayesian perspective. Genetics 180, 977–993 (2008). 18780740 10.1534/genetics.108.092221 PMC2567396

[b43] Fischer M. C., Foll M., Excoffier L. & Heckel G. Enhanced AFLP genome scans detect local adaptation in high-altitude populations of a small rodent (*Microtus arvalis*). Molecular Ecology 20, 1450–1462 (2011). 21352386 10.1111/j.1365-294X.2011.05015.x

[b44] Beaumont M. A. & Nichols R. A. Evaluating loci for use in the genetic analysis of population structure. Proceedings of the Royal Society B 263, 1619–1626 (1996).

[b45] Lewontin R. & Krakauer J. Distribution of gene frequency as a test of theory of selective neutrality of polymorphisms. Genetics 74, 175–195 (1973). 4711903 10.1093/genetics/74.1.175 PMC1212935

[b46] Letunic I., Yamada T., Kanehisa M. & Bork P. iPath: interactive exploration of biochemical pathways and networks. Trends in Biochemical Sciences 33(3), 101–103 (2008). 18276143 10.1016/j.tibs.2008.01.001

[b47] Yamada T., Letunic I., Okuda S., Kanehisa M. & Bork P. iPath2.0: interactive pathway explorer. Nucleic Acids Research 39(suppl 2), W412–W415 (2011). 21546551 10.1093/nar/gkr313 PMC3125749

