# Supplementary material for: Characterization of genome-wide SNPs for the water flea Daphnia pulicaria generated by genotyping-by-sequencing (GBS)
Source: Sci Rep. 2016 Jun 27;6:28569. doi: 10.1038/srep28569 (PMC4921830; doi:10.1038/srep28569)
Supplement: Supplementary Information [file srep28569-s2.pdf]

:Manuscript information:

Manuscript title: Characterization of genome-wide SNPs for the water flea *Daphnia pulicaria* generated by genotyping-by-sequencing (GBS)

Manuscript code: SREP-15-34629

Manuscript author list: Joaquín Muñoz, Anurag Chaturvedi, Luc De Meester, Lawrence J Weider

Supplementary figure:

Manuscript title: Characterization of genome-wide SNPs for the water flea Daphnia pulicaria generated by genotyping-by-sequencing (GBS)

Manuscript code: **SREP-15-34629**

Manuscript author list: Joaquín Muñoz, Anurag Chaturvedi, Luc De Meester, Lawrence J Weider

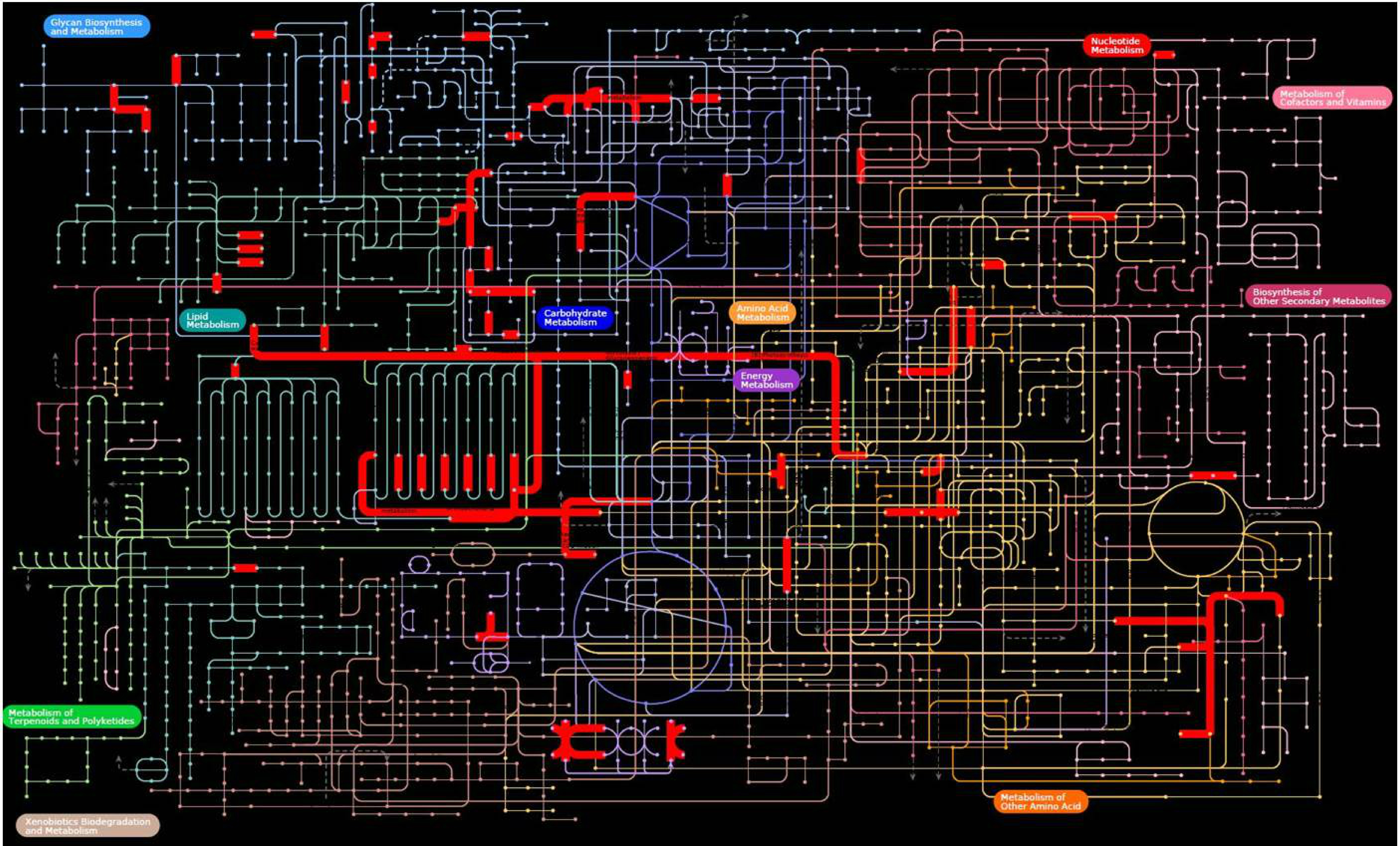

Appendix 2: Informative box including step by step the pipeline followed in this study for confident SNP call mapping on the *Daphnia pulex* reference genome

**Informative box: Ten steps to follow**
• Step #1: FastqToTagCountPlugin – Starting from a raw FASTQ file, sequences/reads are converted to tags (i.e., *barcoded reads collapsed into a set*)
perl /tassel_dir/run_pipeline.pl -fork1 -FastqToTagCountPlugin -i /work_dir/fastq/ -k /work_dir/key/daphnia_key.txt -e ApeKI -o /work_dir/tagCounts/ -endPlugin -runfork1 | tee daphnia_log.txt
• Step #2: Merge multiple tags – Reads are typically the result of running multiple sequencing lanes (or multiple flow cells). Here, we merged each 'TagCount' file of each lane into a 'masterTagCount' list keeping only tags with a total count greater than or equal that the specified in option '-c' (i.e., *minimum number of times a tag must be present*). We used '-c 5'
perl /tassel_dir/run_pipeline.pl -fork1 -MergeMultipleTagCountPlugin -i /work_dir/tagCounts/ -c 5 -o /work_dir/mergedTagCounts/myMasterGBSTags.cnt -endPlugin -runfork1 | tee daphnia_log.txt
• Step #3: Generate fastq file for mapping – The masterTagCount file was converted into FASTQ file for mapping tags onto the reference genome
perl daphnia_log.txt -fork1 -TagCountToFastqPlugin –i /work_dir/mergedTagCounts/myMasterGBSTags.cnt -c 5 -o /work_dir/mergedTagCounts/myMasterGBSTags.fastq -endPlugin -runfork1 | tee daphnia_log.txt
• Step #4: Download reference genome – We downloaded the *Daphnia pulex* reference file (in .fasta format) from wfleabase website. In addition, we replaced scaffold number for appropriate use in TASSEL
wget wfleabase.org/genome/Daphnia_pulex/current/fasta/dpulex-all-chromosome-jgi060905.fasta.gz<br>zcat dpulex-all-chromosome-jgi060905.fasta.gz | sed 's/scaffold_//g' >dpulex-all-chromosome-jgi060905.renamed.fasta
• Step #5: Map the fastq file from above step (i.e., *step #3*) to *Daphnia pulex* genome – This will create a .sam file containing the aligned short reads over the reference genome. To do this, we used Burrows-Wheeler Aligner (BWA)
bwa aln -t 8 /work_dir/genome/dpulex-all-chromosome-jgi060905.renamed.fasta //work_dir/mergedTagCounts/myMasterGBSTags.fastq >daphnia.sai<br>bwa samse /work_dir/genome/dpulex-all-chromosome-jgi060905.renamed.fasta daphnia.sai /work_dir/mergedTagCounts/myMasterGBSTags.fastq >daphnia.sam
• Step #6: Convert sam file to topm file – The alignment (i.e., *sam file*) was converted into a file containing the genomic position of each sequence tag with a unique best alignment (i.e., *topm file*)
perl /tassel_dir/run_pipeline.pl -fork1 -SAMConverterPlugin -i /work_dir/mergedTagCounts/daphnia.sam -o /work_dir/topm/ -endPlugin -runfork1 | tee daphnia_log.txt
• Step #7: Fastq to TBT - The barcode information in the original FASTQ file is used to tally the number of times each tag in the master tag list is observed in each sample/taxon. These counts are stored in a TagsByTaxa (TBT) file
perl /tassel_dir/run_pipeline.pl -fork1 -FastqToTBTPlugin -i /work_dir/fastq/ -k /work_dir/key/daphnia_key.txt -e ApeKI -c 1 -o /work_dir/tbt/ -y -t /work_dir/mergedTagCounts/myMasterGBSTags.cnt -endPlugin -runfork1 | tee daphnia_log.txt
• Step #8: Merge tags by taxa – Here, we merged all TBT files that are too large to fit in memory when they are combined. This new file will keep only presence or absence of a tag in a sample/taxon using a Boolean strategy of the individual counts
perl /tassel_dir/run_pipeline.pl -fork1 -MergeTagsByTaxaFilesPlugin -i /work_dir/tbt/ -o /work_dir/mergedTBT/daphnia.tbt.byte -endPlugin -runfork1 | tee daphnia_log.txt
• Step #9: TBT to VCF – Before merge duplicate SNPs, we converted the merged TBT file to a Variant Call Format (VCF) file. It is a text file storing all the information about the gene sequence variations in a compressed manner (e.g., chromosome/scaffold, position, ID of the variation, quality, filter)
perl /tassel_dir/run_pipeline.pl -fork1 -tbt2vcfPlugin -i /work_dir/mergedTBT/daphnia.tbt.byte -m /work_dir/topm/daphnia.topm -o /work_dir/vcf/ -sC 1 -eC 9080 -ak 3 -mnSLCov 0.0 --mnMAF 0.0 -endPlugin -runfork1 | tee daphnia_log.txt
• Step #10: Merge duplicate SNPs – With this step we expect to find duplicate SNPs coming from the VCF file. SNPs are merged if they have the same pair of alleles producing a new VCF file, which can be easily converted into a HapMap
perl /tassel_dir/run_pipeline.pl -fork1 -MergeDuplicateSNP_vcf_Plugin -i /work_dir/vcf/mergedTBT."chr_number" -o /work_dir/vcf/mergedduplicatevcf."chr_number" -ak 3 -endPlugin -runfork1 | tee daphnia_log.txt
